# Brooding and neuroticism are strongly interrelated manifestations of the phenome of depression

**DOI:** 10.3389/fpsyt.2023.1249839

**Published:** 2023-12-22

**Authors:** Asara Vasupanrajit, Michael Maes, Ketsupar Jirakran, Chavit Tunvirachaisakul

**Affiliations:** ^1^Department of Psychiatry, Faculty of Medicine, Chulalongkorn University, Bangkok, Thailand; ^2^Ph.D. Program in Mental Health, Department of Psychiatry, Faculty of Medicine, Chulalongkorn University, Bangkok, Thailand; ^3^Sichuan Provincial Center for Mental Health, Sichuan Provincial People’s Hospital, School of Medicine, University of Electronic Science and Technology of China, Chengdu, China; ^4^Key Laboratory of Psychosomatic Medicine, Chinese Academy of Medical Sciences, Chengdu, China; ^5^Cognitive Impairment and Dementia Research Unit, Faculty of Medicine, Chulalongkorn University, Bangkok, Thailand; ^6^Cognitive Fitness and Biopsychological Technology Research Unit, Faculty of Medicine, Chulalongkorn University, Bangkok, Thailand; ^7^Department of Psychiatry, Medical University of Plovdiv, Plovdiv, Bulgaria; ^8^Research Institute, Medical University of Plovdiv, Plovdiv, Bulgaria; ^9^Kyung Hee University, Seoul, Republic of Korea; ^10^Center of Excellence for Maximizing Children's Developmental Potential, Department of Pediatric, Faculty of Medicine, Chulalongkorn University, Bangkok, Thailand

**Keywords:** major depression, affective disorders, stress, precision psychiatry, suicide

## Abstract

**Introduction:**

We found that neuroticism may be identified as a subclinical manifestation of the phenome of depression, comprising depressive and anxiety symptoms, and suicidal behaviors. Rumination is positively associated with depression and neuroticism and may mediate the effects of neuroticism on depression. This study aimed to determine whether rumination or its components, including brooding or reflection, mediate the effects of neuroticism on depression or, alternatively, whether both neuroticism and rumination are manifestations of the phenome of depression.

**Methods:**

This study recruited 74 depressed subjects and 44 healthy controls. The depression group was split into groups with high versus low brooding scores. We used partial least squares (PLS) to examine mediation effects.

**Results:**

We found that brooding and reflection scores are significantly higher in depressed patients than in controls. Patients with higher brooding scores have increased severity of depression, anxiety, insomnia, neuroticism, and current suicidal ideation as compared with patients with lower brooding scores and controls. There is a strong positive association between rumination, and neuroticism, depression, anxiety, and lifetime and current suicidal behaviors. PLS analysis shows that brooding does not mediate the effects of neuroticism on the depression phenome because no discriminant validity could be established between neuroticism and brooding, or between neuroticism and brooding and the depression phenome. We were able to extract one validated latent vector from brooding and neuroticism, insomnia, depression, anxiety, and current suicidal behaviors.

**Conclusion:**

Overall, this study supports the theory that rumination and neuroticism are reflective manifestations of the phenome of depression.

## Introduction

Depression is one of the most common mental disorders and a significant global burden (1–3). With an average onset age of 24 years, the 12-month prevalence and lifetime prevalence of depression are approximately 5.9% and 11.1%, respectively (4). Individuals with depression exhibit a variety of symptoms, including loss of interest, sadness, anxious mood, inability to concentrate, sleep problems, low self-esteem, and suicidal ideation or behaviors (5–7).

Neuroticism is a personality trait that refers to a tendency for unpleasant emotions and increased emotionality, impulsivity, fear, and anger (8). Recent evidence suggests that high levels of neuroticism increase the risk of depression and anxiety symptoms (9, 10). A common psychological explanation is that the chronic negative emotional trait associated with neuroticism may result in an aberrant response to environmental stressors, thereby increasing the likelihood of hopelessness and, consequently, depression (11). Moreover, a number of studies demonstrate that depressive patients have cognitive impairments in attention and working memory that result in a selective focus on negative stimuli (12–15) and exposure to unavoidable stressors that activate depressogenic thoughts (12, 13, 16).

Rumination is a cognitive process characterized by a pattern of activities and/or thoughts that inhibit mood and impede problem-solving, instrumental activity, and social support (17–19). Rumination is associated with the development and persistence of the symptoms of several mental health conditions, such as depression, anxiety, insomnia, obsessive-compulsive disorder (OCD), eating disorders, somatic symptom disorder, post-traumatic stress disorder (PTSD), and substance use disorders (19, 20). Rumination consists of two factors: brooding and reflection (21). In contrast to brooding, which consists of dwelling on unpleasant feelings over and over again, reflective thinking comprises active cognitive problem-solving or adaptive thinking, with the goal of alleviating such feelings (21).

Earlier research showed a substantial link between ruminating and neuroticism (22). Moreover, ruminating, especially brooding, may contribute to the onset of depression and predict suicidal behaviors (22–25) and may mediate the effects of neuroticism on depression (26–28). Andrews and Thomson (16) have proposed that depressed patients show the tendency to repeat their thoughts and analyze their problems, thereby disrupting concentration processes and inducing anhedonia and depression. Rumination may impede working memory processes by retaining outdated information and focusing on unpleasant information, resulting in memory errors that may function as long-term stressors (29). Therefore, ruminative traits may lead to a negative selection bias (29) that makes neurotic individuals more susceptible to the impact of adverse life experiences and depressive symptoms (30, 31). In addition, rumination is reported to mediate the effects of depressive mood on sleep quality, suggesting that a psychological mechanism mediates the effects on insomnia. Pre-sleep rumination also predicts poor sleep quality, including delayed sleep onset in individuals with depressive symptoms (32).

Recent research has shown that a latent vector may be extracted from neuroticism (a trait) and the acute phenome (symptomatology of severe depression), which consists of the severity of depression and anxiety, as well as suicidal thoughts (SI) and attempts (SA) (9, 33). This suggests that neuroticism is a subclinical expression of the phenome of major depressive disorder (9, 33). Nevertheless, no studies have examined whether ruminating may mediate the effects of neuroticism on the phenome of depression, including suicidal behaviors and sleep disorders, or alternatively, that rumination is a feature of the phenome of depression.

Hence, the aims of this study were to determine whether (a) rumination mediates the effects of neuroticism on the phenome of depression, including suicidal behaviors and sleep disorders; or (b) rumination and neuroticism are, as depression, anxiety, insomnia, and suicidal ideation, reflective indicators of the same underlying latent construct, namely, the depression phenome. Toward this end, we use partial least squares (PLS) analysis, which combines regression, mediation, and factor analysis.

## Materials and methods

### Participants

The *a priori* sample size was estimated using G*Power version 3.1.9.4. According to a power analysis, the minimum sample size should be *n* = 111 when using an ANCOVA with an effect size of *f* = 0.3, alpha = 0.05, power = 0.8, number of groups = 3, and covariates = 6. Therefore, between November 2021 and February 2023, we recruited 118 Thai-speaking university students who were aged between 18 and 35 years and of both sexes. The students attended different faculties of the Chulalongkorn University, Bangkok, Thailand. The depressed students (*n* = 74) were recruited at the Department of Psychiatry, King Chulalongkorn Memorial Hospital, Bangkok, Thailand. They all complied with the DSM-5-TR (5) criteria for major depressive disorder (MDD) and showed a Hamilton Depression Rating Scale (HAM-D) (34) score > 7, indicating that no patients in remission were included. The patient group was divided into high and low brooding groups using the median-split method. Exclusion criteria were as follows: axis 1 psychiatric disorders, including schizophrenia, alcohol or substance use disorders, psycho-organic disorders, anxiety disorders, autism, bipolar disorder, and schizoaffective disorder; axis 2 diagnoses including antisocial and borderline personality disorder; individuals with high risk of committing suicide; current medical illness including immune and neuro-inflammatory disorders, such as diabetes type 1, chronic kidney disease, endocrine or autoimmune disorders, psoriasis, lupus erythematosus, Parkinson’s disease, Alzheimer’s disease, multiple sclerosis, and stroke. Pregnant and lactating female students were excluded from participating. We excluded those who had a lifetime diagnosis of moderate-to-severe COVID-19 infection and those who had suffered from mild COVID-19 symptoms the month prior to inclusion in the study. Furthermore, subjects with possible long COVID (or other infectious disease) symptoms including chronic fatigue, cough, and fever were excluded. Forty-four healthy controls were included if their lifetime history of psychiatric disorders and suicidality was negative and the HAM-D (34) score ≤ 7. They were recruited through word of mouth and online advertisements and were matched to the patients for age, sex, and number of education years. The students were consecutively admitted to participate in the study; we excluded 3 students (1 in the control group, and 2 in the MDD group) based on the exclusion criteria.

This is a case–control study reviewed and approved by the Institutional Review Board (IRB) of the Faculty of Medicine, Chulalongkorn University, Bangkok, Thailand (IRB No. 351/63). All participants gave written informed consent prior to the study.

### Measures

We used a semi-structured interview to obtain socio-demographic data comprising age, sex, relationship status, year of education, current smoking, lifetime history of COVID-19, and family history of mental health, including major depressive disorder, bipolar disorder, anxiety, and psychotic and suicidal histories. The Ruminative Response Scale (RRS) (35) has been used to assess rumination. The RRS comprises 22 items rated on a 4-point Likert scale ranging from almost never (1) to almost always (4). This study used the Thai version (36), which shows good internal consistency (α = 0.90) and content validity (CVI = 0.95). The International Personality Item Pool-NEO (IPIP-NEO) (37) was developed based on the Big Five personality traits and comprises 50 phrases describing an individual’s behavior, which were selected as proxies for the broad domain scores of NEO-PI-R (8). This study used the Thai version translated by Yomaboot and Cooper (38). The Thai version was examined using exploratory and confirmatory factor analyses, which revealed an acceptable fit and a five-factor model consisting of eight items for neuroticism (N), three items for extraversion (E), five items for agreeableness (A), six items for openness (O), and eight items for consciousness (C). Internal consistency for this 30-item version was acceptable to be good (Cronbach’s alpha for N = 0.83, E = 0.76, A = 0.37, O = 0.67, C = 0.73).

This study used the HAM-D (34) and the Beck Depression Inventory-II (BDI-II) (39) to evaluate the symptoms and severity of depression. We used the HAM-D Thai version, translated by Lotrakul et al. (40). The BDI-II (39) is a self-rating questionnaire comprising 21 items rated on a 4-point Likert scale ranging from not present (0) to severe (3). The Thai version of the BDI-II was translated by Mungpanich (41) and has excellent validity and reliability. The State–Trait Anxiety Inventory (STAI) (42) is a self-rating questionnaire to assess state anxiety and comprises 20 items ranging from 1 (not at all) to 4 (mostly). The total score ranges from 20 to 80 with higher scores indicating greater anxiety. The Thai version was translated by Iamsupasit and Phumivuthisarn (43) and has good internal consistency with a Cronbach’s alpha coefficient ranging from 0.86 to 0.92. The Columbia—Suicide Severity Rating Scale (C-SSRS) (44) is a semi-structured interview that was used to evaluate lifetime (until 1 month prior to inclusion of the participants) and current (within 1 month) suicidal behaviors (SB). The C-SSRS is divided into four constructs including the severity of suicidal ideation, the intensity of suicidal ideation, suicidal behaviors (self-harm and attempts), and the lethality subscale. The Thai version was provided by The Columbia Lighthouse Project (45).

The Insomnia Severity Index (ISI) (46, 47) is a self-rating questionnaire to assess the severity of sleep problems. The ISI comprises 7-point Likert-scale items ranging from 0 (no problem) to 4 (very severe problems). The total score of 0–7 is interpreted as the absence of insomnia; 8–14 as sub-threshold insomnia; 15–21 as moderate insomnia; and 22–28 as severe insomnia. The ISI Thai version is provided by the Mapi Research Trust (48) The body mass index (BMI) was computed as weight (kg) divided by height (m) squared.

### Statistical analyses

Using Pearson’s product–moment correlation coefficients, the analysis investigated the correlations between continuous variables. Analysis of variance (ANOVA) was utilized to investigate the associations between diagnostic groups and clinical data. A contingency table analysis (*χ*^2^ test) was used to evaluate statistical associations between categorical variables. Principal component analysis (PCA) was utilized to reduce the number of items into a single PC score, which could then be utilized in other statistical analyses. Using the Kaiser–Meyer–Olkin (KMO) test for sample adequacy, which is considered adequate when >0.6, and Bartlett’s sphericity test, factorability was evaluated. The first PC is admitted only if the variance explained (VE) is greater than 50% and all loadings on the first PC are greater than 0.66. Using SmartPLS path analysis (49), we evaluated the causal relationships between neuroticism, rumination (brooding and reflection), and phenome of depression. The latter was conceptualized as a latent vector extracted from the severity of depression (HAM-D and BDI-II), anxiety (STAI), insomnia (ISI score), and current SB. Neuroticism was conceptualized as a latent vector extracted from 6 IPIP-NEO items, brooding as a latent vector extracted from 14 RRS, and reflection as a latent vector extracted from 3 RRS items. Other indicators were entered as singular indicators (age and sex). Complete PLS analysis was performed when the outer and inner models satisfied predefined quality criteria, namely: (a) all loadings on the latent vectors are greater than 0.7 at *p* < 0.001; (b) the model fit SRMR is less than 0.08; (c) all latent vectors have adequate composite reliability (> 0.7), Cronbach’s alpha (> 0.7), and rho A (> 0.8) scores, with an average variance extracted (AVE) greater than 0.50; (d) the models are not incorrectly specified as reflective models, as confirmed by confirmatory tetrad analysis. The discriminant validity of the constructs was checked using the heterotrait–monotrait ratio (HTMT) with a cutoff value of 0.85. Using 5,000 bootstrap samples, a complete PLS analysis was conducted to calculate specific indirect, total indirect, and total direct path coefficients (with exact *p*-values). The above PLS analysis which examined the effects of neuroticism and brooding on the phenome of depression was the primary statistical analysis.

## Results

### Results of PC analysis

We were not able to extract one validated PC from all RRS items. However, we were able to extract one validated PC from items 1, 2, 3, 4, 5, 6, 9, 10, 13, 14, 15, 16, 17, 18, 19, and 22, as shown in Table 1. As all items on this validated PC score highly on brooding items, it was labeled as “PC_brooding.” Electronic Supplementary File (ESF), Supplementary Table S1 lists all RRS items. We were also able to extract one PC from items 7, 11, 20, and 21, as shown in Table 1. As these 4 items score highly on self-reflection, it was labeled as “PC_reflection.” Using PC_brooding and PC_reflection, we constructed a composite, which is a weighted composite of rumination.

**Table 1:** Results of principal component (PC) analysis and construction of brooding and reflection principal components (PCs) based on the items of the Ruminative Response Scale (RRS) rating scale.

PC_brooding		PC-reflection	
Variables	Loading	Variables	Loading
RRS1	0.791	RRS7	0.851
RRS2	0.754	RRS11	0.788
RRS3	0.842	RRS20	0.856
RRS4	0.793	RRS21	0.701
RRS5	0.722		
RRS6	0.771		
RRS9	0.762		
RRS10	0.567		
RRS13	0.611		
RRS14	0.720		
RRS15	0.731		
RRS16	0.811		
RRS17	0.854		
RRS18	0.846		
RRS19	0.815		
RRS22	0.733		
KMO = 0.933		KMO = 0.720	
*χ*^2^ = 1357.987 (df = 120), *p* < 0.001		*χ*^2^ = 176.048 (df = 6), *p* < 0.001	
VE = 58.01%		VE = 64.27%	

KMO, The Kaiser–Meyer–Olkin Test; VE, Variance explained; *χ*^2^, Bartlett’s test of sphericity. Supplementary Table S1 shows the explanation of the RRS items.

In accordance with previous research (50), we made new constructs reflecting lifetime (LT) and current SB, including SI and SA. ESF, Supplementary Tables S2, S3 show how we constructed LT_SI, LT_SA, LT_SB, current_SI, current_SA, and current_SB. To make a new comprehensive phenome index, we extracted the first PC from the total HAM-D (loading = 0.918), BDI-II (loading = 0.922), STAI (loading = 0.745), ISI (loading = 0.767), and current_SB (loading = 0.758) scores (KMO = 0.830, Bartlett’s *χ*^2^ = 346.103, df = 10, *p* < 0.001, explained variance = 68.229%). This validated PC was labeled “current_phenome.”

### Socio-demographic and clinical data

To display differences in depression patients with and without high brooding scores, we have binned the patient group into two subgroups based on the median values of PC_brooding. Table 2 shows the socio-demographic and basic clinical features of the participants. There were no significant differences in age, sex, BMI, number of education years, and current smoking frequency between depression with high and low PC_brooding scores and controls. There are no significant differences in depression ICD10 subtypes, duration of depression, medication status, previous mild COVID-19 infection, and psychiatric and suicide history among family members between both patient groups. To augment the efficacy of antidepressants, five participants were treated with mood stabilizers.

**Table 2:** Demographic and clinical data of depressive patients with high versus low brooding scores and healthy controls (HC).

Variables	HC (*n* = 44)	Depression—low brooding (*n* = 37)	Depression—high brooding (*n* = 37)	F/*χ*^2^/FET	df	*p*
Age (years)	23.5 (3.2)	22.5 (3.5)	22.2 (2.4)	1.99	2/115	0.141
Sex (male/female)	7/37	6/31	6/31	0.00	2	0.999
Education (years)	16.6 (1.5)	16.1 (3.0)	15.5 (2.2)	2.57	2/115	0.081
BMI (kg/m^2^)	22.3 (3.6)	22.4 (4.8)	22.1 (5.4)	0.03	2/115	0.966
Relationship status (single/boy or girlfriends)	27/17	16/21	14/23	7.27	2	0.122
Current smoking (yes/no)	1/43	3/34	3/34	1.84	2	0.432
Lifetime COVID-19 history (yes/no)	14/30^c^	12/25	4/33^a^	6.07	2	0.048
ICD-10 diagnosis (F32/F33/F34)	–	34/0/3	33/3/1	4.02	2	0.134
Duration of depression (months)	–	21.0 (16.6)	27.6 (26.7)	1.63	1/72	0.206
Psychiatric medication						
Antidepressant (yes/no)	–	34/3	36/1	FET	–	0.615
Benzodiazepine (yes/no)	–	15/22	22/15	2.65	–	0.104
Antipsychotic (yes/no)	–	7/30	7/30	0.00	1	1.000
Mood stabilizer (yes/no)	–	3/34	2/35	FET	–	1.000
Psychiatric history in family						
Depression (yes/no)	–	7/30	5/32	0.40	–	0.754
Bipolar (yes/no)	–	1/36	0/37	FET	–	1.000
Anxiety (yes/no)	–	2/35	1/36	FET	–	1.000
Psychotic (yes/no)	–	1/36	0/37	FET	–	1.000
Suicide history in family (yes/no)	–	4/33	3/34	FET	–	1.000
Total RRS score	44.2 (12.4)^b,c^	59.6 (7.6)^a,c^	75.6 (6.7)^a,b^	110.81	2/115	<0.001
PC_brooding	−0.888 (0.796)^b,c^	−0.024 (0.502)^a,c^	1.080 (0.278)^a,b^	114.47	2/115	<0.001
PC_reflection	−0.817 (0.838)^b,c^	0.270 (0.675)^a,c^	0.701 (0.747)^a,b^	43.33	2/115	<0.001

Results are shown as mean (S.D.); F, results of analysis of variance; *χ*^2^, analysis of contingency tables; FET, Fisher’s exact probability test; RRS, The Ruminative Response Scale; PC, principal component; ^a,b,c^pairwise comparisons using LSD (*p* < 0.05), i.e., ^a^means significant different from HC, ^b^means significant different from depression—low brooding, ^c^means significant different from depression—high brooding.

### Clinical features of patients with current suicidal ideation

Table 3 shows the clinical features of healthy controls and both patient groups. The HAM-D, BDI-II, STAI, ISI, and current phenome scores were significantly different between the three study groups and increased from controls → low brooding → high brooding groups. LT and current SI, SA, and SB were significantly higher in both patient groups than controls. Nevertheless, current_SI was significantly different between the three study groups with the highest values in those with high brooding. Furthermore, there was a significant difference in three dimensions of personality, namely, higher neuroticism, lower agreeableness, and lower conscientiousness, in both depressive groups as compared with controls. The most significant personality trait associated with depression was neuroticism. In analogy with Jirakran et al. (9), this study will, therefore, focus on neuroticism.

**Table 3:** Features of depressive patients with high versus low brooding scores and healthy controls (HC).

Variables	HC (*n* = 44)	Depression—low brooding (*n* = 37)	Depression—high brooding (*n* = 37)	*F*	df	*p*
Symptoms						
HAM-D	2.4 (2.0)^b,c^	13.9 (5.2)^a,c^	18.9 (6.8)^a,b^	118.86	2/115	<0.001
BDI-II	6.8 (6.5)^b,c^	21.1 (11.6)^a,c^	32.6 (10.7)^a,b^	72.64	2/115	<0.001
STAI	46.6 (14.1)^b,c^	56.6 (8.6)^a,c^	64.1 (6.3)^a,b^	27.06	2/115	<0.001
ISI	5.6 (4.3)^b,c^	11.2 (6.2)^a,c^	14.9 (6.2)^a,b^	33.33	2/115	<0.001
Current_phenome	−0.994 (0.442)^b,c^	0.256 (0.647)^a,c^	0.952 (0.632)^a,b^	119.43	2/114	<0.001
Lifetime (LT) suicide						
LT_SI	−1.033 (0.590)^b,c^	0.583 (0.631)^a^	0.645 (0.595)^a^	102.51	2/115	<0.001
LT_SA	−0.700 (0)^b,c^	0.315 (0.994)^a^	0.518 (1.133)^a^	KWT	–	<0.001
LT_SB	−0.936 (0.319)^b,c^	0.485 (0.805)^a^	0.628 (0.873)^a^	64.59	2/115	<0.001
Current suicide						
Current_SI	−0.848 (0)^b,c^	0.232 (0.966)^a,c^	0.777 (0.873)^a,b^	KWT	–	<0.001
Current_SA	−0.329 (0)^b,c^	0.173 (1.124)^a^	0.219 (1.332)^a^	KWT	–	0.020
Current_SB	−0.673 (0)^b,c^	0.231 (1.068)^a^	0.569 (1.081)^a^	KWT	–	<0.001
All SB	−0.932 (0.197)^b,c^	0.416 (0.834)^a^	0.692 (0.881)^a^	65.66	2/115	<0.001
Personality						
Neuroticism	17.02 (5.29)^b,c^	27.19 (5.57)^a,c^	33.16 (4.13)^a,b^	100.93	2/115	<0.001
Agreeableness	22.20 (2.60)^b,c^	19.16 (3.23)^a^	19.68 (3.69)^a^	10.89	2/115	<0.001
Conscientiousness	27.25 (4.47)^b,c^	24.84 (5.16)^a,c^	22.35 (5.22)^a,b^	9.80	2/115	<0.001
Extraversion	9.36 (3.00)	9.38 (2.74)	7.89 (3.47)	3.45	2/115	0.058
Openness	18.89 (2.70)^b^	20.32 (1.87)^a^	19.73 (3.06)	3.14	2/115	0.047

Results are shown as mean (S.D.); F, results of analysis of variance; PC, principal component; HAM-D, Hamilton Depression Rating Scale; BDI, The Beck Depression Inventory; STAI, State Trait Anxiety Assessment; ISI, The Insomnia Severity Index; SB, suicidal behavior; SI, suicidal ideation; SA, suicidal attempt; ^a,b,c^pairwise comparisons using LSD (*p* < 0.05), i.e., ^a^means significant different from HC, ^b^means significant different from depression—low brooding, ^c^means significant different from depression—high brooding; KWT: Kruskal–Wallis test.

### Intercorrelation matrix

**Figure 1:**
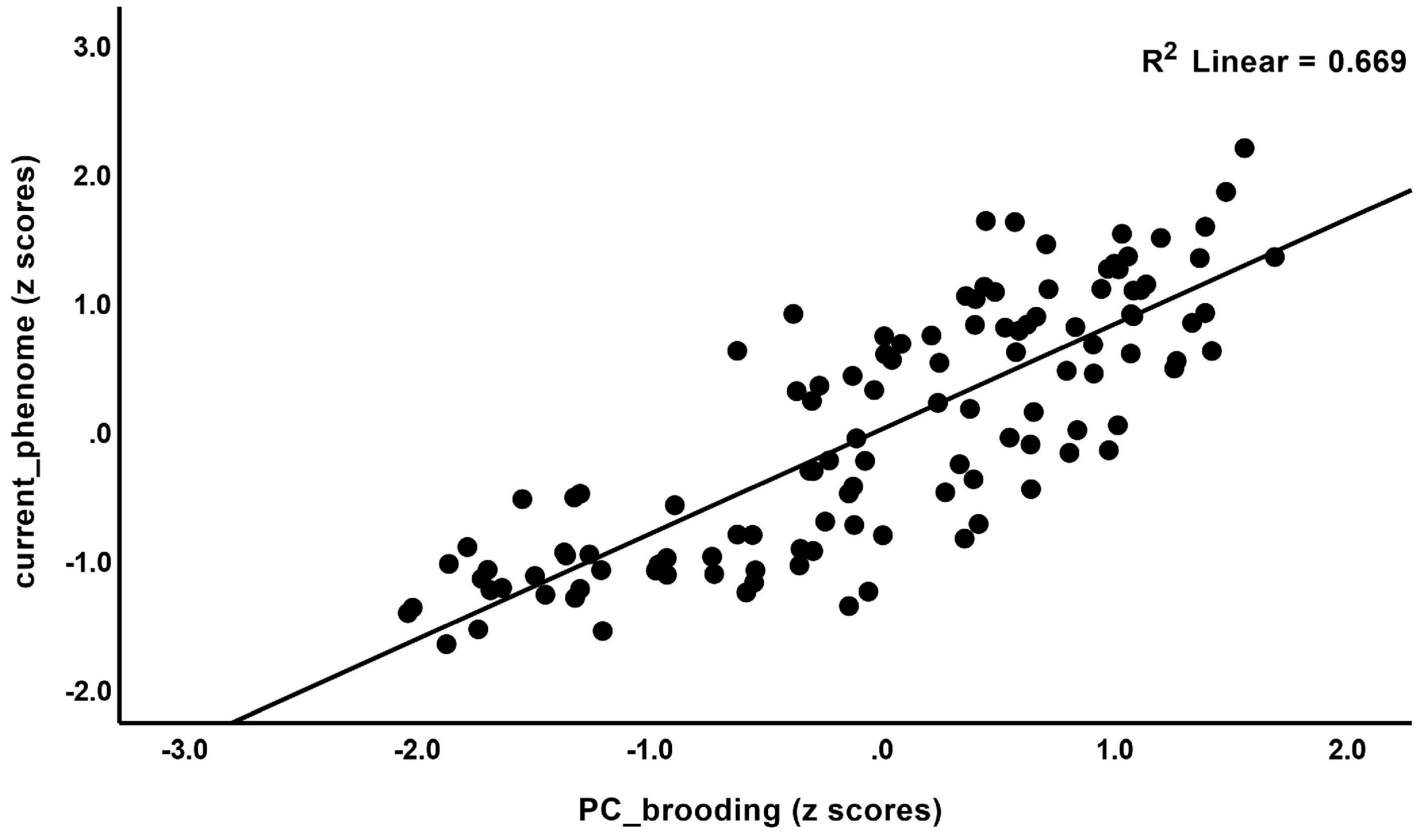
Partial regression of current_phenome on PC_brooding (after allowing for the effects of age, sex, and education).

**Figure 2:**
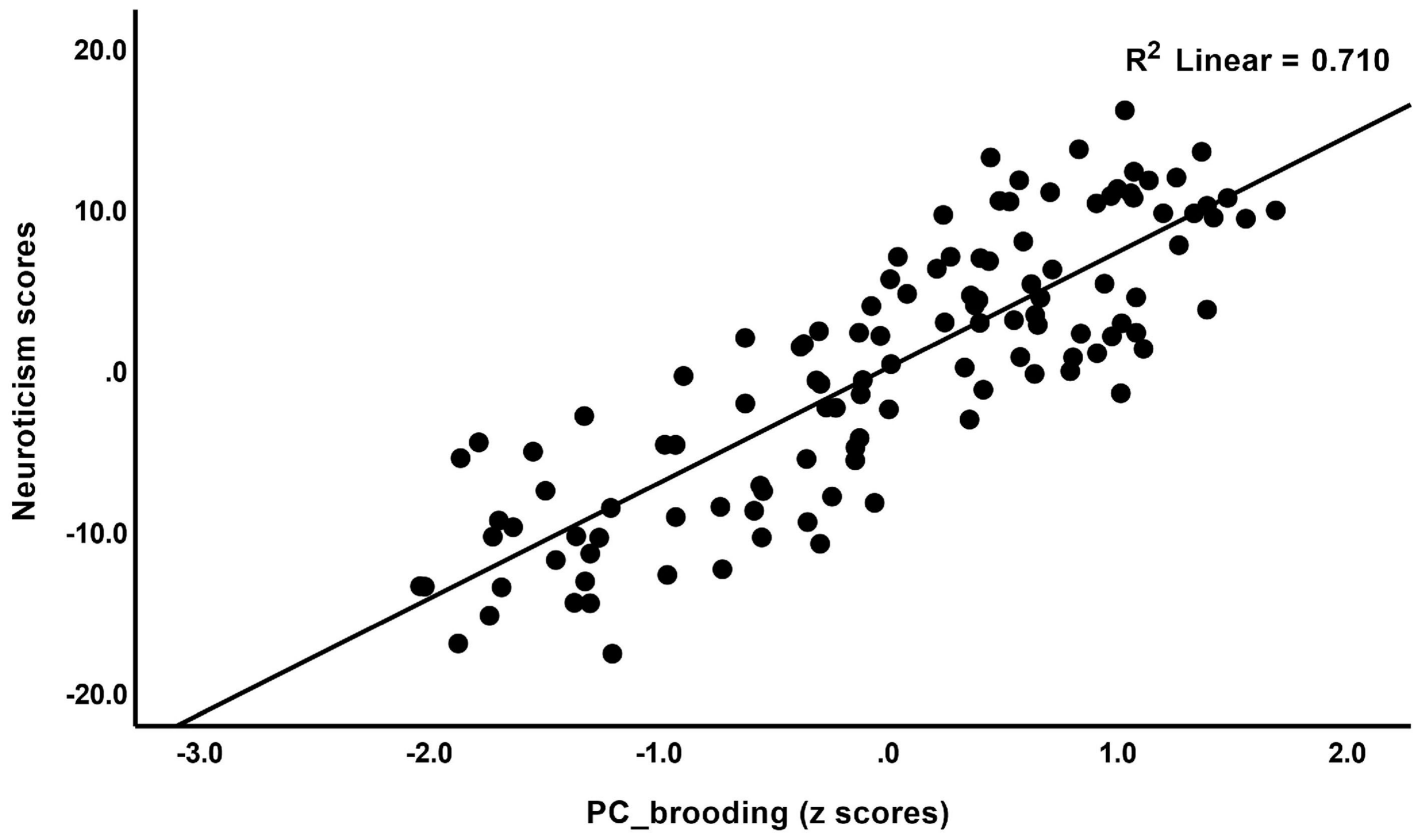
Partial regression of neuroticism on PC_brooding.

**Figure 3:**
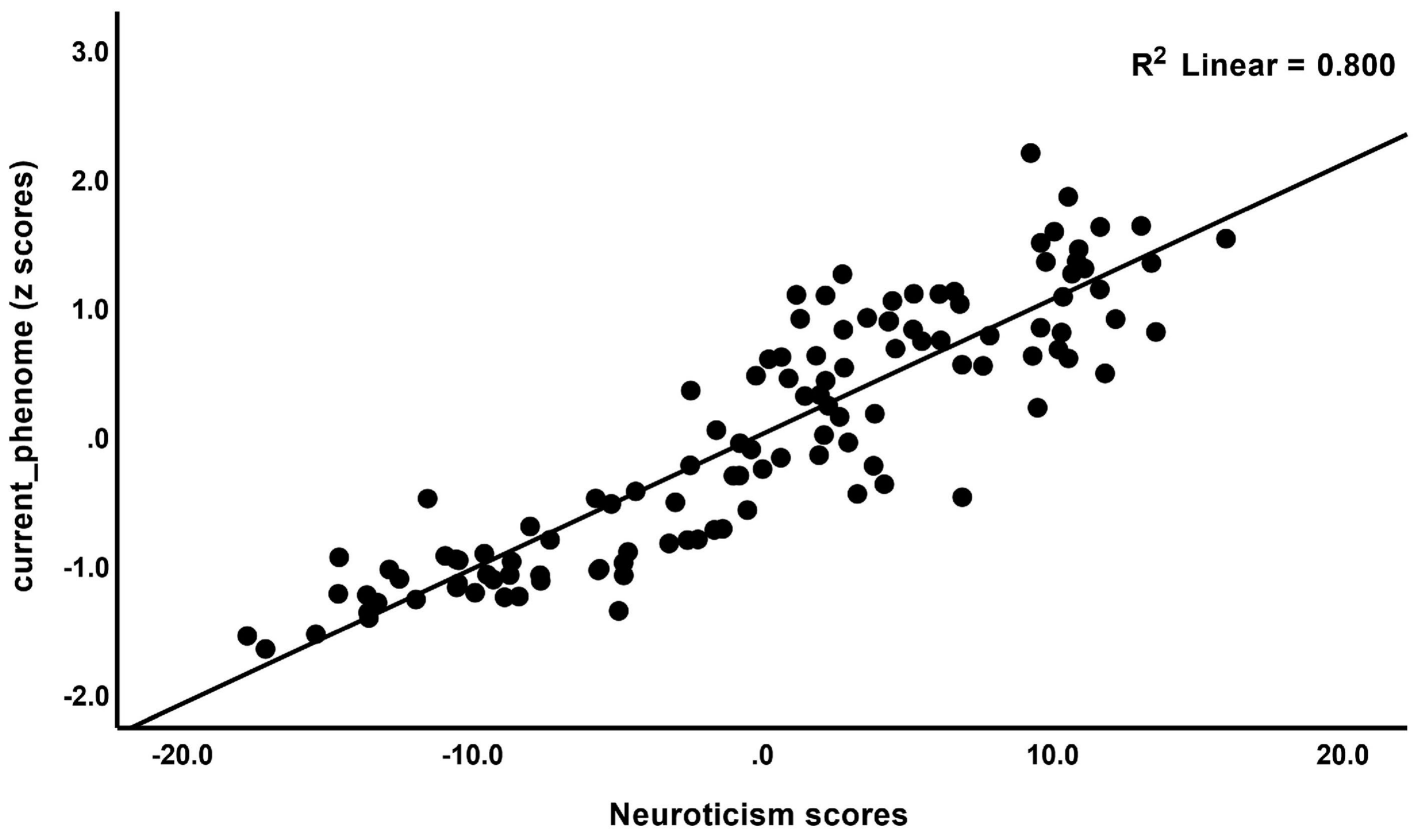
Partial regression of current_phenome on neuroticism.

The correlations between rumination and clinical data, including current phenome, HAM-D, BDI-II, STAI, ISI, LT and current SI, SA, SB, and PC_neuroticism are shown in Table 4. There were positive relationships between rumination, as well as PC_brooding, and all clinical variables (*r* = 0.4–0.8, *p* ≤ 0.001). Rumination, both brooding and reflection, showed strong positive associations with the current phenome and neuroticism. Figure 1 shows the partial regression of current_phenome on PC_brooding (after controlling for the effects of age, sex, and education). Figure 2 shows the partial regression of neuroticism on PC_brooding and Figure 3 shows the partial regression of current_phenome on neuroticism. There was a significant correlation between rumination and PC_brooding, and LT and current SA. PC_reflection showed positive relationships with all these clinical variables, except current SA.

**Table 4:** Correlation matrix between rumination, brooding, and reflection and other clinical features of depression.

Variables	Rumination composite	PC_brooding	PC_reflection
Rumination	1	–	–
PC_brooding	0.921 (<0.001)	1	–
PC_reflection	0.921 (<0.001)	0.696 (<0.001)	1
Current_phenome	0.790 (<0.001)	0.826 (<0.001)	0.629 (<0.001)
HAM-D	0.705 (<0.001)	0.706 (<0.001)	0.593 (<0.001)
BDI-II	0.733 (<0.001)	0.771 (<0.001)	0.578 (<0.001)
STAI	0.656 (<0.001)	0.761 (<0.001)	0.447 (<0.001)
ISI	0.632 (<0.001)	0.605 (<0.001)	0.559 (<0.001)
Lifetime (LT) suicide			
LT_SI	0.666 (<0.001)	0.647 (<0.001)	0.579 (<0.001)
LT_SA	0.392 (<0.001)	0.399 (<0.001)	0.324 (<0.001)
LT_SB	0.572 (<0.001)	0.565 (<0.001)	0.488 (<0.001)
Current suicide			
Current_SI	0.572 (0.001)	0.594 (<0.001)	0.460 (<0.001)
Current_SA	0.199 (0.030)	0.242 (0.008)	0.125 (0.179)
Current_SB	0.790 (<0.001)	0.478 (<0.001)	0.334 (<0.001)
All SB	0.587 (<0.001)	0.601 (<0.001)	0.480 (<0.001)
Neuroticism	0.870 (<0.001)	0.837 (<0.001)	0.517 (<0.001)

Results of Spearman’s rank-order correlation calculations; PC, principal components; HAM-D, Hamilton Depression Rating Scale; BDI, The Beck Depression Inventory; STAI, State Trait Anxiety Assessment; ISI, The Insomnia Severity Index; SB, suicidal behavior; SI, suicidal ideation; SA, suicidal attempt; RRS, The Ruminative Response Scale.

As some items of the HAM-D and BDI-II may reflect rumination, we have examined two depression PCs, which do not contain any rumination or neuroticism items, and their associations with brooding and neuroticism.

The first was constructed as the first PC extracted from 3 HAM-D items (depression, loss of interest, and suicide) and 4 BDI-II items (sadness, loss of pleasure, suicide, and loss of interest) (KMO = 0.870, variance explained = 64.51%, all loadings >0.744), labeled “PC_depression.” The second PC was constructed as the first PC extracted from 3 HAM-D items (somatic anxiety, gastro-intestinal somatic, and general somatic) and 2 BDI-II items (loss of energy and fatigue) (KMO = 0.758, variance explained = 59.86%, all loadings >0.678), labeled as “PC_somatic.” Consequently, we have examined whether PC_depression, PC_somatic, PC_neuroticism, and PC_brooding are reflective manifestations of the same latent construct. We were able to derive one validated PC (KMO = 0.821, VE = 82.12%) from the PC_depression (0.884), PC_somatic (0.915), PC_neuroticism (0.935), and PC_brooding (0.890) that loaded highly on this component. Neuroticism and brooding were significantly correlated with PC_depression (*r* = 0.750 and *r* = 0.666, respectively, both: *p* < 0.001) and PC_somatic (*r* = 0.798 and *r* = 0.7224, respectively, both: *p* < 0.001).

### Results from PLS analysis

**Figure 4:**
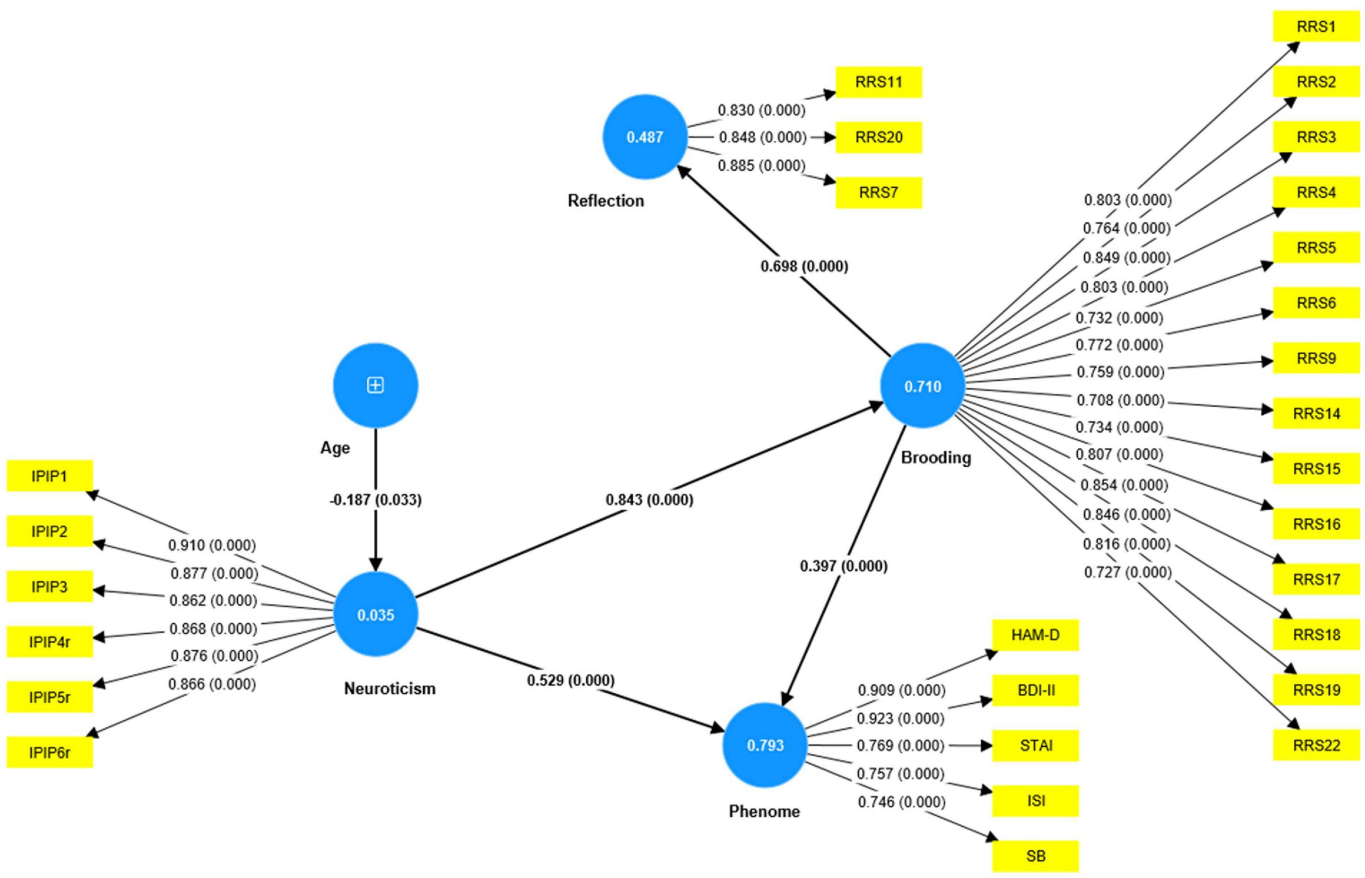
Results of PLS analysis. Shown are the significant paths, including the path coefficients (with exact *p* value) of the inner model, and loadings (with *p* values) of the outer model. Figures in blue circles indicate explained variance. Age was entered as a single indicator (denoted as +); neuroticism, phenome, brooding, and reflection were entered as latent vectors extracted from their manifestations. IPIP, The International Personality Item Pool-NEO (IPIP-NEO); HAM-D, Hamilton Depression Rating Scale; BDI-II, The Beck Depression Inventory; STAI, State Trait Anxiety Assessment; ISI, The Insomnia Severity Index; SB, suicidal behaviors; RRS, The Ruminative Response Scale.

Based on the theories described in the Introduction, we constructed the first PLS model as shown in Figure 4. The depression phenome was conceptualized as a latent vector extracted from HAM-D, BDI-II, STAI, ISI, and current SB scores. Neuroticism was a latent vector extracted from six IPIP-NEO neuroticism subdomain items. We also constructed brooding and reflection latent vectors and entered the items shown in Table 2 as indicators. The neuroticism, brooding, and reflection latent vectors predicted the phenome, and brooding could mediate the effects of neuroticism on the phenome. According to psychological theories that distress, and brooding may lead to reflection, PC_brooding was related to PC_reflection. Age was employed as a single explanatory variable that could predict all latent vectors. The model in Figure 4 shows only the significant paths. The model fit quality was adequate with SRMR = 0.066. Neuroticism, brooding, reflection, and the phenome revealed adequate construct validity and convergence with (a) an average variance extracted (AVEs) of 0.769, 0.617, 0.730, and 0.680, respectively; (b) Cronbach’s alpha of 0.940, 0.952, 0.817, and 0.880, respectively; and (c) composite reliability of 0.952, 0.957, 0.890, and 0.913, respectively. All loadings of the outer model were greater than 0.7. We found that 79.3% of the phenome variance was explained by neuroticism and brooding. There were specific indirect effects of neuroticism on the phenome (*t* = 5.26, *p* < 0.001) and on reflection (*t* = 11.63, *p* < 0.001), which were mediated by brooding. Age showed a specific indirect effect on the phenome that was mediated by neuroticism (*t* = 2.03, *p* = 0.043). There were significant total effects of neuroticism (*t* = 37.19, *p* < 0.001) and brooding (*t* = 5.25, *p* < 0.001) on the phenome, whereas age showed an inverse total effect (*t* = −2.13, *p* = 0.033).

**Figure 5:**
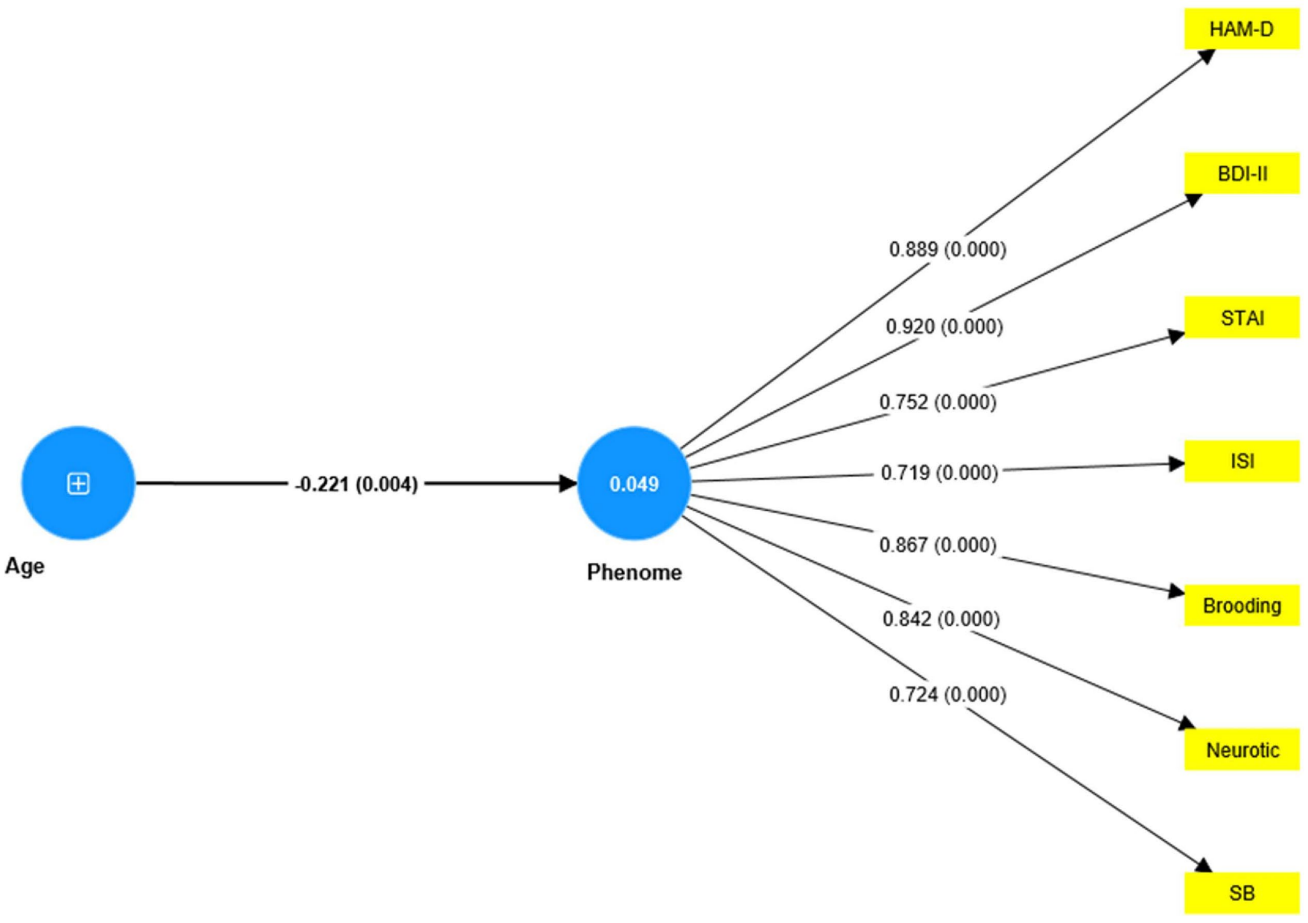
Results of PLS analysis. Shown are the significant paths, including the path coefficients (with exact *p* value) of the inner model, and loadings (with *p*-values) of the outer model. Figures in blue circles indicate the explained variance. Age was entered as a single indicator (denoted as +); the Phenome was entered as a latent vector extracted from HAM-D, Hamilton Depression Rating Scale; BDI-II, The Beck Depression Inventory; STAI, State Trait Anxiety Assessment; ISI, The Insomnia Severity Index; SB, suicidal behavior.

Nevertheless, this first model is not adequate because no significant discriminatory validity could be established between the phenome and either neuroticism (HTMT = 0.942) and brooding (HTMT = 0.915), and between neuroticism and brooding (HTMT = 0.884). Interestingly, discriminant validity could be established between reflection and brooding (HTMT = 0.776), reflection and neuroticism (HTMT = 0.669), and reflection and the phenome (HTMT = 0.747). Therefore, we have tried to combine both neuroticism and brooding into the same phenome latent vector (see Figure 5). Age was introduced to the model as an explanatory variable of the phenome. This model fit was adequate with SRMR = 0.051. The overall phenome latent vector showed adequate validity with an AVE of 0.672, Cronbach’s alpha of 0.919, and composite reliability of 0.971. All loadings were greater than 0.7. PC_reflection showed a loading of only 0.641 and thus could not be included.

## Discussion

### Increased brooding and reflection in depression

The first main finding of this study is that rumination scores, including both brooding and reflection, were significantly higher in depressed patients than in healthy controls, with brooding having a stronger association than reflection. According to Treynor et al. (21), brooding is a maladaptive fixation on distress symptoms, whereas reflection is an adaptive way to engage in cognitive problem-solving. Previous research has shown that brooding is a significant predictor of depression over time, whereas there is less evidence that reflection has the same effect (51–53). Previous research has shown that rumination and introspection are associated with the development of depressive symptoms and that rumination may predict the onset of depression (18, 53, 54). Other studies demonstrated a correlation between rumination and the severity (22), duration (22), and recovery (55) of depression. According to Koster et al. (13), rumination indicates a lack of cognitive control to divert attention away from negative self-references. Owens and Gibb (54) found that ruminating was associated with enhanced selective attention to depression-related information, resulting in an increased risk of depression, onset, and relapse. It is possible that ruminations interfere with reward systems, causing depressed individuals to repeatedly focus on negative outcomes, such as negative self-evaluations and judgments, resulting in a diminished ability to generate and experience positive effects (13, 56).

### Brooding as a symptom of depression

The second major finding of our study is that no discriminatory validity was established between rumination and the phenome of depression, and that one latent vector could be extracted from ruminating, depressive and anxiety symptoms, insomnia, and suicidal behaviors. In contrast, previous research examined rumination as a predictor (22, 57), mediator (26, 28), and consequence (57) of depression. In a longitudinal study, Whisman et al. (57) discovered that rumination has a bidirectional association with changes in depressive symptoms in community adults, both as a risk factor and a consequence. Ruminating is a complete and partial mediator between depression and anxiety in adolescents and adults, respectively, and may increase the risk of comorbidity between these affective disorders (58).

Our PLS model, however, shows that brooding (assessed as a trait) is a manifestation of the phenome of depression, which therefore should be regarded as the cause of its manifestations, including increased brooding (assessed as a trait), the severity of depression and anxiety (assessed as state features over the last 1/2 weeks), insomnia (assessed as a state feature over the last 2 weeks), and suicidal behaviors (assessed as a state feature over the last month). According to Treynor et al. (21), the RRS was created as a self-report of ruminations about depressive symptoms that partially correlate with BDI items. In actuality, the RRS scale items reveal self-ratings of rumination regarding depressive symptoms based on BDI items, such as exhaustion, motivation, concentration difficulties, personal failings, and regret (35, 39). Overall, our study indicates that increased brooding about depressive symptoms should be considered a manifestation of the phenome of depression, just like anxiety, loss of interest, suicidal ideation, insomnia, and cognitive deficits in attention, executive functions, and memory (59). Moreover, once present, brooding can exacerbate the symptoms of depression by interfering with cognitive processes and reward processing, thereby increasing the flow of negative information, and decreasing the capacity to engage in positive responses (13, 54, 56).

### Brooding and neuroticism as manifestations of depression

The third major finding of this study is that our initial PLS model (Figure 4) demonstrating that brooding mediates the effects of neuroticism on the depressive phenome could not be validated. Previously, Roelofs et al. (27) and Lyon et al. (26) found that brooding and refection mediate the effects of neuroticism on depressive symptoms. In our study, however, no discriminatory validity could be established between neuroticism and brooding or between neuroticism and the depressive phenome, thereby excluding the possibility that brooding is a mediator. Next, we were able to incorporate neuroticism, brooding, and all phenome features into one single latent vector, indicating that neuroticism and brooding are reflective manifestations of the same underlying latent construct, namely, the depression phenome.

With respect to neuroticism, our findings are consistent with those of Jirakran et al. (9), who demonstrated that a latent vector can be extracted from neuroticism (a personality trait) and state characteristics of the depression phenome in major depressed patients with a longer duration of illness. We found, like Jirakran et al. (9), that neuroticism is the personality trait that is most strongly associated with depression, whereas agreeableness and consciousness (both decreased in depression) are less significant depression-associated characteristics. Numerous studies have documented an association between neuroticism and depression [e.g., (30, 31, 57)]. In cross-sectional and longitudinal studies, neuroticism has been associated with rumination and depressive symptoms. Moreover, du Pont et al. (60) reported that rumination and neuroticism have a shared genetic basis.

Numerous psychological theories could, of course, explain the significant relationships between depression, neuroticism, and rumination. Those with neuroticism and a more ruminative response style, for example, may have abnormalities in reward processes, working memory, and executive functions, which may fuel ruminations about the negative effects of depression and contribute to negative self-evaluations and self-reflections. In addition, this process may result in a greater retention of negative cognition, leading to a decline in problem-solving and interpersonal functioning. Nevertheless, we demonstrated previously that the phenome of depression is strongly influenced by a variety of biological pathways that could lead to neuro-affective toxicity by interfering with affective-cognitive neuronal circuits (61) and thus all phenotypic characteristics of depression. Interestingly, the same biomarkers that underlie the depressive phenome are also strongly associated with neuroticism (62). These findings provide additional evidence that neuroticism is a component of the phenome of depression, which is caused by biological pathways. Future research should investigate whether the same biomarkers are also linked to rumination and brooding. As rumination may be a trait-like cognitive response to distress (17), it is conceivable that the phenome of the acute phase of depression exacerbates this trait, thereby contributing causatively to the phenome of MDD.

## Limitations

This study should be interpreted considering its limitations. Firstly, it would be essential to investigate rumination during the partially remitted and remitted phases of depression, as well as the preclinical phase preceding the onset of depression. The incorporation of depression due to Long COVID may be an intervening factor (63). Nevertheless, we included a few students who had previously suffered from mild acute COVID-19 infection and omitted participants who had suffered from moderate-to-severe COVID-19 infection. As Long-COVID develops in patients with critical COVID-19 infection (63), we minimized bias due to previous COVID-19 infection. It could be argued that the sample size is rather small. Nonetheless, the number of participants included in our study was based on an *a priori* estimate of sample size, and post-hoc power analysis revealed that we worked at a power of 1.0.

It could be argued that the associations established here between depression and brooding and neuroticism may not be independent because some items of the HAM-D and BDI-II reflect brooding and neuroticism. Nevertheless, we have constructed two latent vectors that reflect pure depressive and somatic symptoms, which do not contain any brooding or neuroticism items. As our results show that brooding and neuroticism belong to the same latent construct as these two latent vectors, we may conclude that there are genuine associations between brooding, neuroticism, and depression, and that brooding, neuroticism, and pure depressive and somatic symptoms are all reflective manifestations of the phenome of depression.

Future research should examine associations between depression, neuroticism, and brooding and the harm avoidance and self-directedness dimensions of the Temperament and Character model (64). Previous research found indeed a partial overlap between neuroticism and harm avoidance (65). This is important because Cloninger’s model emphasizes the psychobiological basis of personality traits (64, 66). Future research should identify the role of other independent indicators, such as biomarkers and functional brain connectivity, in relation to rumination, neuroticism, and depression. In this regard, functional magnetic resonance imaging research revealed that rumination, neuroticism, and depression were associated with irregularities in neurocircuitries, such as the default mode and salience networks (67–70).

Furthermore, rumination and neuroticism have been observed in a broad range of psychopathologies (19, 20, 71). Therefore, future research should examine whether the same associations may be observed in, for example, anxiety disorders.

## Conclusion

Overall, this study supports the hypothesis that rumination and neuroticism (both assessed as traits) are manifestations of the depression phenome. The results of this study add to our understanding that brooding could be considered as an important manifestation of the depressive phenome. Therefore, brooding should be incorporated in rating scales that measure the severity of depression and probably also as a diagnostic criterion to make the diagnosis of depression. Future studies should examine whether the biological pathways, which may lead to neuro-affective toxicity in depression, are associated with brooding.

## Data availability statement

The raw data supporting the conclusions of this article will be made available by the authors, without undue reservation.

## Ethics statement

The studies involving humans were approved by The Institutional Review Board of Chulalongkorn University’s institutional ethics board, Bangkok, Thailand. The studies were conducted in accordance with the local legislation and institutional requirements. The participants provided their written informed consent to participate in this study. Written informed consent was obtained from the individual(s) for the publication of any potentially identifiable images or data included in this article.

## Author contributions

AV and MM designed the current study and performed the statistical evaluation. AV contributed to the data collection. All authors contributed to the writing and rewriting of the manuscript, and they have all given their consent for submission of the completed version.
