# Supplementary material for: Brooding and neuroticism are strongly interrelated manifestations of the phenome of depression
Source: Front Psychiatry. 2023 Dec 22;14:1249839. doi: 10.3389/fpsyt.2023.1249839 (PMC10766685; doi:10.3389/fpsyt.2023.1249839)
Supplement: Supplementary file 1 [file Table_1.docx]

**Electronic supplementary file (ESF)**

**Brooding and neuroticism are strongly interrelated manifestations of the phenome of depression.**

**Running title:** Rumination, neuroticism, and depression phenome

(1,2) Asara Vasupanrajit*, (1-9) Michael Maes*, (1,2,10) Ketsupar Jirakran, (1,2,5) Chavit Tunvirachaisakul

* Joint first authorship.

1. Department of Psychiatry, Faculty of Medicine, Chulalongkorn University, Bangkok, Thailand
2. Ph.D. Program in Mental Health, Department of Psychiatry, Faculty of Medicine, Chulalongkorn University, Bangkok, Thailand
3. Sichuan Provincial Center for Mental Health, Sichuan Provincial People’s Hospital, School of Medicine, University of Electronic Science and Technology of China, Chengdu, China
4. Key Laboratory of Psychosomatic Medicine, Chinese Academy of Medical Sciences, Chengdu, China
5. Cognitive Impairment and Dementia Research Unit, Faculty of Medicine, Chulalongkorn University, Bangkok, Thailand
6. Cognitive Fitness and Biopsychological Technology Research Unit, Faculty of Medicine Chulalongkorn University, Bangkok, Thailand
7. Department of Psychiatry, Medical University of Plovdiv, Plovdiv, Bulgaria
8. Research Institute, Medical University of Plovdiv, Plovdiv, Bulgaria
9. Kyung Hee University, Seoul, Korea
10. Center of Excellence for Maximizing Children's Developmental Potential, Department of Pediatric, Faculty of Medicine, Chulalongkorn University, Bangkok, Thailand

**Corresponding author:**

Prof. Dr Michael Maes, M.D., PhD.

Department of Psychiatry,

Faculty of Medicine, Chulalongkorn University,

Bangkok, 10330, Thailand

And

Prof. Dr. Michael Maes, M.D., Ph.D.

Sichuan Provincial Center for Mental Health

Sichuan Provincial People’s Hospital,

School of Medicine,

University of Electronic Science and Technology of China

Chengdu 610072, China

**Co-corresponding author:**

Chavit Tunvirachaisakul, M.D., PhD.

Department of Psychiatry,

Faculty of Medicine, Chulalongkorn University,

Bangkok, 10330, Thailand

**ESF, Table 1.** Description of rumination items from The Ruminative Response Scale (RRS)

| **Items** | **Description** |
| --- | --- |
| RRS1 | Think: how lonely you are. |
| RRS2 | Think: "I won't be able to do my job if I don't snap out of this" |
| RRS3 | Think: how exhausted and weak you feel. |
| RRS4 | Think: how difficult it is to focus. |
| RRS5 | Think: "What have I done to deserve this?" |
| RRS6 | Think: how helpless and unmotivated you feel. |
| RRS7 | Analyze recent occurrences to try to figure out why you're depressed. |
| RRS8 | Think: how you seem not to be feeling anything anymore. |
| RRS9 | Think: “Why can't I get started?” |
| RRS10 | Think: "Why do I always react like this?" |
| RRS11 | Go away by yourself and reflect on why you feel this way. |
| RRS12 | Make a list of your thoughts and examine them. |
| RRS13 | Think: about recent circumstance in which you wish things had gone differently. |
| RRS14 | Think: "If I keep feeling like this, I won't be able to concentrate." |
| RRS15 | Think: "Why do I have problems that others do not?" |
| RRS16 | Think: "How come I can't handle things better?" |
| RRS17 | Think: how depressed you are. |
| RRS18 | Think: about all your weaknesses, foibles, faults, and errors. |
| RRS19 | Think: how you don't feel like undertaking anything. |
| RRS20 | Analyze your personality to try to figure out why you're depressed. |
| RRS21 | Go somewhere alone to reflect on your thoughts. |
| RRS22 | Think: how furious you are with yourself. |

**ESF, Table 2.** Descriptions of suicide items from The Columbia–Suicide Severity Rating Scale (C-SSRS)

| **Items** | **Descriptions** | **Construction1** | **Construction2** |
| --- | --- | --- | --- |
| SI_LT1 | CSSR Lifetime wish to be death | Lifetime suicide ideation (SI) | Suicide behaviors (SB) |
| SI_LT2 | CSSR Lifetime non-specific active suicidal thought |  |  |
| SI_LT3 | CSSR Lifetime suicidal thoughts with methods |  |  |
| SI_LT4 | CSSR Lifetime suicidal intent |  |  |
| SI_LT5 | CSSR Lifetime suicidal intent with plan |  |  |
| SI_LT6 | CSSR Lifetime frequency of suicide ideation |  |  |
| SI_LT7 | CSSR Lifetime duration of suicide ideation |  |  |
| SI_LT8 | CSSR Lifetime ability to control suicide ideation |  |  |
| SI_LT9 | CSSR Lifetime deterrent to suicide |  |  |
| SI_LT10 | CSSR Lifetime reason of suicidal ideation |  |  |
| SI_C1 | CSSR Current wish to be death (1 month) | Current suicide ideation (SI)  within 1 month |  |
| SI_C2 | CSSR Current non-specific active suicidal thought (1 month) |  |  |
| SI_C3 | CSSR Current suicidal thoughts with methods (1 month) |  |  |
| SI_C4 | CSSR Current suicidal intent (1 month) |  |  |
| SI_C5 | CSSR Current suicidal intent with plan (1 month) |  |  |
| SI_C6 | CSSR Current frequency of suicide ideation (1 month) |  |  |
| SI_C7 | CSSR Current duration of suicide ideation (1 month) |  |  |
| SI_C8 | CSSR Current ability to control suicide ideation (1 month) |  |  |
| SI_C9 | CSSR Current deterrent to suicide (1 month) |  |  |
| SI_C10 | CSSR Current reason of suicidal ideation (1 month) |  |  |
| SA_LT1 | CSSR Lifetime suicidal behavior actual attempt | Lifetime suicide acts (SA) |  |
| SA_LT2 | CSSR Lifetime total number of suicidal behavior actual attempt |  |  |
| SA_LT3 | CSSR Lifetime nonsuicidal self-injury |  |  |
| SA_LT4 | CSSR Lifetime interrupted attempt |  |  |
| SA_LT5 | CSSR Lifetime total number of interrupted attempts |  |  |
| SA_LT6 | CSSR Lifetime self-interrupted attempt |  |  |
| SA_LT7 | CSSR Lifetime total number of self-interrupted attempt |  |  |
| SA_LT8 | CSSR Lifetime preparatory acts |  |  |
| SA_LT9 | CSSR Lifetime suicidal acts |  |  |
| SA_C1 | CSSR Current suicidal behavior actual attempt (1 month) | Current suicide acts (SA)  within 1 month |  |
| SA_C2 | CSSR Current total number of suicidal behavior actual attempt (1 month) |  |  |
| SA_C3 | CSSR Current nonsuicidal self-injury (1 month) |  |  |
| SA_C4 | CSSR Current interrupted attempt (1 month) |  |  |
| SA_C5 | CSSR Current total number of interrupted attempt (1 month) |  |  |
| SA_C6 | CSSR Current self-interrupted attempt (1 month) |  |  |
| SA_C7 | CSSR Current total number of self-interrupted attempt (1 month) |  |  |
| SA_C8 | CSSR Current preparatory acts (1 month) |  |  |
| SA_C9 | CSSR Current suicidal acts (1 month) |  |  |

**ESF, Table 3.** Principle component (PC) analyses of suicide

| **PC- lifetime SI** | | **PC- current SI** | | **PC- lifetime SA** | | **PC- current SA** | |
| --- | --- | --- | --- | --- | --- | --- | --- |
| **Variables** | **Loading** | **Variables** | **Loading** | **Variables** | **Loading** | **Variables** | **Loading** |
| SI_LT1 | 0.869 | SI_C1 | 0.896 | SA_LT1 | 0.917 | SA_C1 | 0.902 |
| SI_LT2 | 0.880 | SI_C2 | 0.836 | SA_LT4 | 0.793 | SA_C2 | 0.893 |
| SI_LT3 | 0.873 | SI_C3 | 0.832 | SA_LT6 | 0.776 | SA_C6 | 0.592 |
| SI_LT4 | 0.801 | SI_C4 | 0.690 | SA_LT8 | 0.952 | SA_C8 | 0.867 |
| SI_LT5 | 0.702 | SI_C5 | 0.564 | SA_LT9 | 0.954 | SA_C9 | 0.886 |
| SI_LT6 | 0.855 | SI_C6 | 0.886 |  |  |  |  |
| SI_LT7 | 0.871 | SI_C7 | 0.869 |  |  |  |  |
| SI_LT8 | 0.840 | SI_C8 | 0.848 |  |  |  |  |
| SI_LT9 | 0.787 | SI_C9 | 0.792 |  |  |  |  |
| SI_LT10 | 0.799 | SI_C10 | 0.866 |  |  |  |  |
| KMO = 0.892 | | KMO = 0.867 | | KMO = 0.821 | | KMO = 0.714 | |
| Χ^2^ = 1202.750 (df=45), p<0.001*** | | Χ^2^ = 1297.079 (df=45), p<0.001*** | | Χ^2^ = 679.114 (df=10), p<0.001*** | | Χ^2^ = 617.758 (df=10), p<0.001*** | |
| VE=68.783% | | VE=66.232% | | VE=77.791% | | VE=69.984% | |

KMO: The Kaiser-Meyer-Olkin Test; VE: Variance explained; Χ^2^: Bartlett’s test of sphericity.

We extracted the “PC-lifetime SI” from SI_LT item 1, 2, 3, 4, 5, 6, 7, 8, 9, and 10 (KMO=0.892, Bartlett’s χ2=1202.750, df=45, p<0.001, explained variance=68.783%, all loading≥0.7). The “PC-current SI” was computed from SI_C item 1, 2, 3, 4, 5, 6, 7, 8, 9, and 10 (KMO=0.867, Bartlett’s χ2=1297.079, df=45, p<0.001, explained variance=66.232%, all loading≥0.6). The “PC-lifetime SA” was computed from SA_LT item 1, 4, 6, 8, and 9 (KMO=0.821, Bartlett’s χ2=679.114, df=10, p<0.001, explained variance=77.791%, all loading≥0.8). The “PC-current SA” was computed from SA_C item 1, 2, 6, 8, and 9 (KMO=0.714, Bartlett’s χ2=617.758, df=10, p<0.001, explained variance=69.987%, all loading≥0.6). The PC-lifetime SB was conceptualized by PC-lifetime SI and PC-lifetime SA.
